# Exploration of neurometabolic alterations in adolescent patients with bipolar depression and non-suicidal self-injury based on proton magnetic resonance spectroscopy

**DOI:** 10.3389/fpsyt.2024.1474170

**Published:** 2025-01-24

**Authors:** Chengji Wang, Yuan Qu, Xiaoqin Shen, Xiaoxiao Tang, Gaiyu Tong, Meier Wati, Manzeremu Naibi, Cheng Zhang, Shaohong Zou

**Affiliations:** ^1^ Graduate School, Xinjiang Medical University, Urumqi, Xinjiang, China; ^2^ Department of Radiology, People’s Hospital of Xinjiang Uygur Autonomous Region, Urumqi, Xinjiang, China; ^3^ Department of Clinical Psychology, People’s Hospital of Xinjiang Uygur Autonomous Region, Urumqi, Xinjiang, China

**Keywords:** adolescent bipolar depression, non-suicidal self-injury, proton magnetic resonance spectroscopy, neurometabolic, China

## Abstract

**Background:**

Adolescent bipolar depression (ABD) refers to depressive episodes that arise in adolescent patients with bipolar disorder. Its identification and diagnosis are challenging, and it is characterized by a high rate of misdiagnosis and disability. Studies have revealed that patients with ABD are more prone to non-suicidal self-injury (NSSI) compared to those with unipolar depression. However, the neuropathophysiological mechanisms behind NSSI in ABD remain unclear. Therefore, this study employed proton magnetic resonance spectroscopy (^1^H-MRS) technology to investigate the potential relationship between NSSI and neurometabolism in the ventromedial prefrontal cortex (vmPFC) of patients with ABD.

**Methods:**

This study compared brain biochemical metabolism between ABD with and without NSSI. Forty ABD were recruited and divided into groups with (n=21) and without NSSI (n=19). Proton magnetic resonance spectroscopy (^1^H-MRS) was used to detect the ratio of biochemical metabolites in the ventromedial prefrontal cortex (vmPFC) of all patients.

**Results:**

There was no statistically significant difference (P>0.05) in the age, gender, only child status, residential status, education level, age of onset, disease course, family history, and 24-item Hamilton Depression Scale (HAMD) score between patients in the NSSI group and those without NSSI group. The N-acetylaspartate (NAA)/creatine (Cr) of patients with NSSI was lower than that of patients without NSSI, and the difference was statistically significant (*Z=-4.347,P<0.001*). There was no statistically significant difference in choline (Cho)/Cr and myo-inositol (mI)/Cr between the group with NSSI and the group without NSSI (*P*>0.05).There is a positive correlation (*r*=0.703,*P*<0.00625) between Cho/Cr and HAMD scores in patients with NSSI, while there is a varying degree of negative correlation (*r*=-0.605,*P*=0.006;*r*=0.624,*P*=0.004) between mI/Cr and age and onset age in patients without NSSI. There is no correlation (*P*>0.05) between other indicators.

**Conclusion:**

Compared with ABD without NSSI, ABD with NSSI have reduced NAA/Cr metabolism in the vmPFC brain area. The level of membrane phospholipid breakdown metabolism in the vmPFC brain area of ABD with NSSI may be related to the severity of depression. The level of phosphoinositol cycle in the vmPFC brain area of ABD without NSSI may be related to age or onset age. Therefore, further validation was required.

## Introduction

Bipolar disorder (BD) is a chronic, periodic, lifelong, and severe mental disorder. Epidemiological studies have shown that approximately 2.4% of the world’s population is affected by BD (1, 2). BD usually has an onset in adolescence or early adulthood and is a disabling mental disorder associated with high morbidity and premature mortality; this disorder is one of the top ten causes of the global disease burden. Bipolar depression refers to a depressive episode that occurs in individuals with BD, that is, in individuals who have previously experienced depressive or manic episodes (3). It is associated with more serious social functional impairment, reduced life span and self-injury, and increased risk of suicide; it causes more serious functional impairment (4). Bipolar depression is the most common onset polarity in type I and type II BD, with up to 40% of patients initially misdiagnosed with major depressive disorder (MDD) (5). Bipolar depression has been identified as “a major, unresolved, clinical, and public health challenge in contemporary psychiatry” (6).

Adolescent bipolar disorder is a common mental disorder in individuals aged 12-18 years old. Adolescent patients with BD have complex clinical manifestations and varying degrees of BD severity, with more depressive episodes observed in clinical practice; thus, adolescent bipolar disorder is difficult to identify and diagnose and has high rates of misdiagnosis, recurrence, disability, and suicide (7). The diagnosis of BD in the adolescent population is based on the same diagnostic criteria as in adults (8). Research has shown that BD is the fourth leading cause of disability in 15- to 19-year-olds. From 1990 to 2017, the global incidence rate of BD increased by 47.74%, and its disability-adjusted life years (DALYs) increased by 54.4%; patients aged 10-19 years accounted for the largest proportion of cases (9). BD, that starts in adolescence, generally has atypical clinical symptoms. Adolescents with bipolar depression experience more frequent symptoms, and the risks of self-injurious behavior, suicidal ideation, and other factors are significantly increased, with a more severe impact on cognitive function (10).

Non-suicidal self-injury (NSSI), defined as the direct and deliberate destruction of one’s own bodily tissue (e.g., cutting, burning, and hitting oneself) in the absence of suicidal intent and is observed in a variety of mental disorders, such as BD, depressive disorder, and borderline personality disorder. NSSI is an important psychiatric problem, and engaging in NSSI most often begins in adolescence; this behavior is associated with adverse outcomes, including an increased risk of suicide (11, 12). The prevalence of NSSI in patients with mental disorders is high, especially in adolescents, and the prevalence of adolescent NSSI has increased in recent years (13). A meta-analysis found that the global prevalence of NSSI among children and adolescents was 19.5% (14). However, the prevalence of NSSI among 13- to 18-year-old Chinese adolescents was approximately 27.4% (15). Another meta-analysis of longitudinal studies on NSSI showed that the prevalence of NSSI peaks at approximately 15-16 years of age (16). Self-harm is one of the third leading causes of disability among people aged 10-24 years (17). Research has shown that the incidence of self-harm behavior among patients with bipolar depression is higher than that among patients with unipolar depression (18). A history of NSSI more strongly predicts future suicide than a history of attempted suicide, and individuals with a history of NSSI are 30 times more likely to commit suicide than the general population (19). NSSI can persist into adulthood and develop resistance to treatment, which makes it an important issue in psychiatry (20).Diler et al. also found that patients with bipolar depression may exhibit more NSSI (21). Therefore, more attention should be devoted to NSSI in adolescent bipolar depression.

At present, the pathophysiological mechanism of NSSI associated with bipolar depression in adolescents is still unclear. Adult studies have shown that bipolar depression patients with impulsive aggressive behavior (such as NSSI) may exhibit functional, structural, and metabolic changes in the prefrontal cortex (22). Proton magnetic resonance spectroscopy (^1^H-MRS) is a special noninvasive, nonradioactive examination technique used to observe metabolic and biochemical changes in living tissue. It can measure the levels of essential biochemical metabolites in the brain, thereby indirectly indicating the functional status of the brain regions. Neurometabolites detected by ^1^H-MRS mainly include N-acetylaspartate (NAA), choline (Cho), myo-inositol (mI), creatine (Cr), and glutamate and glutamine (Glx). ^1^H-MRS has been a popular research tool in the field of psychiatry in recent years. Huber et al. observed abnormal levels of NAA/Cr, Glx/Cr and mI/Cr in the frontal lobe of pediatric patients with BD (23). Olvera et al. conducted a ^1^H-MRS study on the left dorsolateral prefrontal cortex (DLPFC) in adolescent patients with BD and healthy controls (HCs) and found that NAA/Cr values in the DLPFC region of adolescent patients with BD were lower than those in the HC group, which may indicate neuronal dysfunction in this region (24). Some studies have shown an association of prefrontal and thalamic dysfunction with abnormal NAA and Cho metabolism in adolescent NSSI patients (25). However, to date, there have been no ^1^H-MRS studies of adolescents with bipolar depression and NSSI.

The ventromedial prefrontal cortex (vmPFC) is closely associated with the regulation of emotions, motivation, learning, attention, as well as social and behavioral decision-making in humans. It is regarded as a pivotal region in the neurobiology of BD, influencing the modulation of emotions and behavior (22). Therefore, this study compared the neurometabolic differences in the vmPFC of adolescent bipolar depression patients with and without NSSI using ^1^H-MRS, providing a reference for exploring its pathophysiological mechanisms.

## Methods

### Participants

Forty-nine adolescent patients with bipolar depression were selected from the clinic or the hospital in the Department of Clinical Psychology of the People’s Hospital of Xinjiang Uygur Autonomous Region in China from May 2022 to May 2023. Among them, 5 patients experienced physical discomfort prior to the examination and did not undergo the examination, thus failing to obtain the ratio of vmPFC neural metabolites. Additionally, 4 patients did not maintain a static head position during the examination and exhibited head movement, leading to incomplete ratios of biochemical metabolites being obtained. Consequently, 40 BD patients were ultimately included in this study. Within one month prior to entering this study, one subject had taken the mood stabilizer lithium carbonate, with the continuous use of lithium carbonate lasting less than one week. In addition, we confirmed that none of the participants had taken any medications that might significantly affect the levels of neurometabolites, such as N-acetylcysteine. All participants were between 12 and 18 years old.

Before the adolescents and their parents decided whether to enter our study, we explained the purpose, content, and procedures of this study to the adolescents and their parents and promised that the results of the study would only be used for academic research. The adolescents and their parents voluntarily decided whether to participate in the study. If they agreed to participate, they signed an informed consent form. In addition, patients had the right to discontinue the study at any point during the study period. Questionnaire data were collected during one-on-one interviews by trained researchers in a quiet room in the ward; the researchers provided verbal and written explanations and obtained informed consent from the patients. Patients completed the survey questionnaire by themselves. If patients had questions about the questionnaire, researchers helped them to understand the question by informing them of the original intention of the question in a nonjudgmental manner. In addition, quality control procedures were implemented during the research process. These involved intensive researcher training, detailed on-site explanations, continuous feedback, and independent supervision of supervisors and on-site researchers. We collected questionnaires and conducted on-site inspections to see if data were missing on any items.

### Collection of clinical data and assessment of patients

Demographic and clinical information of participants was collected through custom-designed case data sheets, including age, sex, age at onset, disease duration, residential location, family history, education level, presence of NSSI and its special circumstances.

The criteria for patient enrolment in this study included the following: (1) met the Diagnostic and Statistical Manual of Mental Disorders, fifth edition (DSM-5), criteria for bipolar disorder(currently experiencing a depressive episode that has lasted for 2 weeks or more); (2) clinically diagnosed by two experienced psychiatric physicians (deputy chief or chief physicians); (3) aged between 12 and 18 years, with no restrictions on sex; (4) were right-handed; (5) had a Young Mania Rating Scale (YMRS) (26) total score <7 and a 24-item Hamilton Depression Scale (HAMD) (27)total score>20; and (6) provided informed consent to participate in the study. The exclusion criteria were as follows: (1) a history of organic brain disease, serious somatic disease (especially endocrine diseases that can cause mental disorders), or severe brain injury and coma; (2) presence of other mental disorders diagnosed according to the DSM-5 criteria; (3) substance use disorder; (4) severe suicidal tendencies requiring hospitalization; (5) a history of electroconvulsive therapy in the past 1 month; (6) claustrophobia; or (7) contraindications for MRI.

We used the diagnostic criteria for NSSI provided by the DSM-5 to determine the presence of NSSI. All interviewers were psychiatrists who received standardized training in clinical and NSSI diagnoses before participating in the survey. They also received two hours of training on the objectives and procedures of the investigation. Finally, based on the DSM-5 diagnostic criteria and structured interviews with two trained psychiatrists, subjects diagnosed with NSSI were placed in the NSSI group, while the rest of subjects were placed in the non-NSSI group.

There were 21 patients with NSSI, including 8 males (38.1%) and 13 females (61.9%). Their ages ranged from 13 to 16 years, the duration of disease (BD) ranged from 9 to 28 months, and the age of onset of BD ranged from 12 to 16 years. There were 19 patients in the group without NSSI, including 9 males (47.4%) and 10 females (52.6%), whose age ranged from 13 to 17 years, the duration of disease (BD) ranged from 12 to 16 months, and the age of onset of BD ranged from 13 to 16 years.

### Image acquisition and data processing

Both MRI and ^1^H-MRS were performed on an Ingenia 3.0T MRI scanner (Ingenia, Philips Healthcare, Netherlands) with a 15-channel phased array head coil. Subjects were instructed not to consume coffee, cigarettes or alcohol within 24h before the examination, and their temperature was taken to exclude patients with fever. Before scanning, the subjects were instructed to sit and rest for half an hour, during which time researchers communicated with them to ensure that the subjects would fully cooperate with scanning procedures. During the scan, subjects lay in a supine position and wore earphones to reduce noise, while foam pads were used to stabilize their heads.

A 3D T1-weighted (T_1_W) fast spoiled gradient recalled (FSPGR) MRI sequence was used for the anatomical localization of MRS. T2-weighted (T_2_W) [repetition time (TR)=3,000 ms, echo time (TE) = 95ms], T_1_W-IR [TR=2,000ms, TE=20ms, inversion time (TI)=800ms], and T_2_W fluid attenuated inversion recovery (FLAIR) [TR=8000 ms, TE=270ms, TI=2,000ms] sequences were routinely conducted. The parameters were as follows: voxel size=0.8×0.8×6mm^3^, 18 slices, field of view (FOV)=24cm. The three sequences had the same scanning positions to exclude organic brain lesions. After determining the absence of organic lesions, ^1^H-MRS was performed, and the anatomical location of the vmPFC was determined by an experienced technologist to ensure a consistent position. All views of interest (VOI) avoided sulci or cerebrospinal fluid. The size of a single voxel was 20×20×20mm^3^.The acquisition parameters were as follows: TE=35ms; TR=2,000ms; average (superposition) number of signals (NSA)=96.

To avoid the interference of factors outside the spectrum voxel VOI on the spectral line quality, the saturation band was manually placed adjacent to the VOI. In the three cross-sectional localization images, it was critical to ensure that the air, bone, fat or blood vessels surrounding the VOI were covered by the saturation bands to avoid impacts on the spectral lines of the spectrum. When the full width at half maxima of the water peak did not meet the requirements of <10, we adjusted the position of the VOI and saturation bands repeatedly and then carried out prescanning. The accuracy of the location of the VOI and saturation bands was judged by prescanning results: (1) the chemical shift-selecting saturation method was used to optimize the water suppression during prescanning to ensure that the percentage of water suppression was more than 99%, and (2) the full width at half maxima of the water peak was <10 Hz. In addition, even if there was an influence of the water peak, it could also be eliminated by the water suppression postprocessing steps. The acquisition time of the ^1^H-MRS sequence was 5 min.

The acquired spectral scanning data were transferred to an ISP workstation, and Spectro View software (ISP7.0, Philips Healthcare, Netherlands) was used for spectral postprocessing. The basic processing of spectral data mainly included residual water peak removal, signal wake correction, spectral line interpolation and Fourier transformation, spectral line phase correction, baseline level adjustment, the selection of peak frequency position and line width setting, and peak Gaussian fitting. NAA was located at 2.02 ppm, Cho at 3.20 ppm, Cr at 3.05 ppm and mI at 3.56 ppm. The concentration of Cr in the brain remains relatively stable, thus it is often used as a reference for the levels of other neurometabolites. Specifically, results are presented in the form of ratios, yielding more stable outcomes compared to raw concentrations. Therefore, after the above spectral locations were determined, the areas under the peaks of NAA, Cho, mI and Cr in the vmPFC were measured, and the NAA/Cr, Cho/Cr and mI/Cr ratios were calculated to understand the changes in metabolites in the vmPFC. An experienced chief radiologist in the MR department determined the stability of the spectral lines and performed the imaging data analysis.

### Statistical analysis

SPSS 27.0 software was used for statistical analysis. Measurement data conforming to a normal distribution are described by the mean ± standard deviation $\left(\bar{x}\pm \text{s}\right)$ , and an independent-sample t test was used for intergroup comparisons. The measurement data with a nonnormal distribution are described by the median (interquartile interval), and comparisons between groups were conducted with the Mann−Whitney U test. Classification data are described using frequencies (percentages), and intergroup comparisons were made with the Pearson χ^2^ test. Depending on whether the data conformed to a normal distribution, Pearson correlation analysis or Spearman correlation analysis was used to determine the correlation between two variables. Bonferroni method was used to correct multiple comparisons, and P<0.05/comparison times was considered statistically significant. The difference between the two groups was statistically significant (P < 0.05).

## Results

### Demographic characteristics of the participants

There were no significant differences between the two groups in age, gender, only child status, residence, education, age of onset, disease duration, family history and HAMD (*P>0.05*) (**Table 1**).

**Table 1:** Demographic and clinical data of adolescent patients with bipolar depression with and without NSSI.

	NSSI (*n*=21)	Non-NSSI (*n*=19)	*Z*/*t/χ^2^*	*P*
^a^Age (years)	15.05 ± 1.83	15.63 ± 1.98	0.970	0.338
Sex			0.351	0.554
Female	13 (61.9)	10 (52.6)		
Male	8 (38.1)	9 (47.4)		
Only child status			0.474	0.491
No	10 (47.6)	7 (36.8)		
Yes	11 (52.4)	12 (63.2)		
Residential location			0.186	0.666
Rural	8 (38.1)	6 (31.6)		
Urban	13 (61.9)	13 (68.4)		
Education level			3.576	0.167
Primary school	3 (14.3)	2 (10.5)		
Middle school	13 (61.9)	7 (36.8)		
High school	5 (23.8)	10 (52.6)		
^a^Age of onset (years)	14.29 ± 1.82	14.79 ± 1.78	-0.883	0.383
^b^Duration of illness (months)	23.00 (9.50-28.00)	15.00 (12.00-16.00)	-0.651	0.515
Family history			0.301	0.583
Yes	6 (28.6)	4 (21.1)		
No	15 (71.4)	15 (78.9)		
^b^HAMD score	25.00 (21.00-27.00)	24.00 (21.00-25.00)	-1.052	0.293

NSSI, Non-suicidal self-injury.

a Independent-samples t test.

b Mann−Whitney U test.

### ^1^H-MRS data in the vmPFC of adolescent patients with bipolar depression with and without NSSI

The NAA/Cr ratio of patients in the NSSI group was lower than that in the non-NSSI group, and the difference was statistically significant (*Z*=-4.347,*P<0.001*). There were no statistically significant differences in the Cho/Cr or mI/Cr ratios between the NSSI group and the non-NSSI group (*P>0.05*) (**Table 2**).

**Table 2:** Comparisons of proton magnetic resonance spectroscopy data in the vmPFC of adolescent patients with bipolar depression with and without NSSI.

	NSSI (*n*=21)	Non-NSSI (*n*=19)	*Z*/*t*	*P*
NAA/Cr ratio	1.38 (1.25-1.57)	1.67 (1.62-1.75)	-4.347	<0.001
Cho/Cr ratio	0.66 (0.56-0.75)	0.68 (0.60-0.72)	-0.650	0.516
mI/Cr ratio	0.47 ± 0.15	0.52 ± 0.16	1.057	0.297

NSSI, Non-suicidal self-injury; vmPFC, ventromedial prefrontal cortex.

NAA, N-acetylaspartate; Cho, choline; mI, myo-inositol; Cr, creatine.

Mann–Whitney U test.

### Correlations between biochemical metabolite ratios and clinical variables

**Figure 1:**
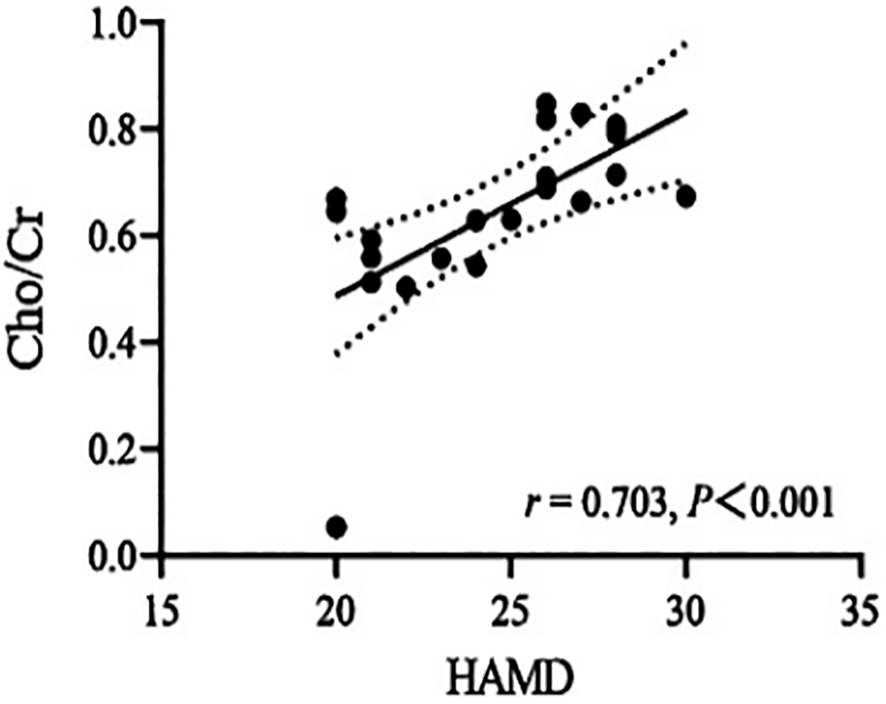
Correlations of Cho/Cr ratios in the ventromedial prefrontal cortex (vmPFC) with HAMD in the NSSI group in which the band with dotted lines shows 95% confidence interval.

**Figure 2:**
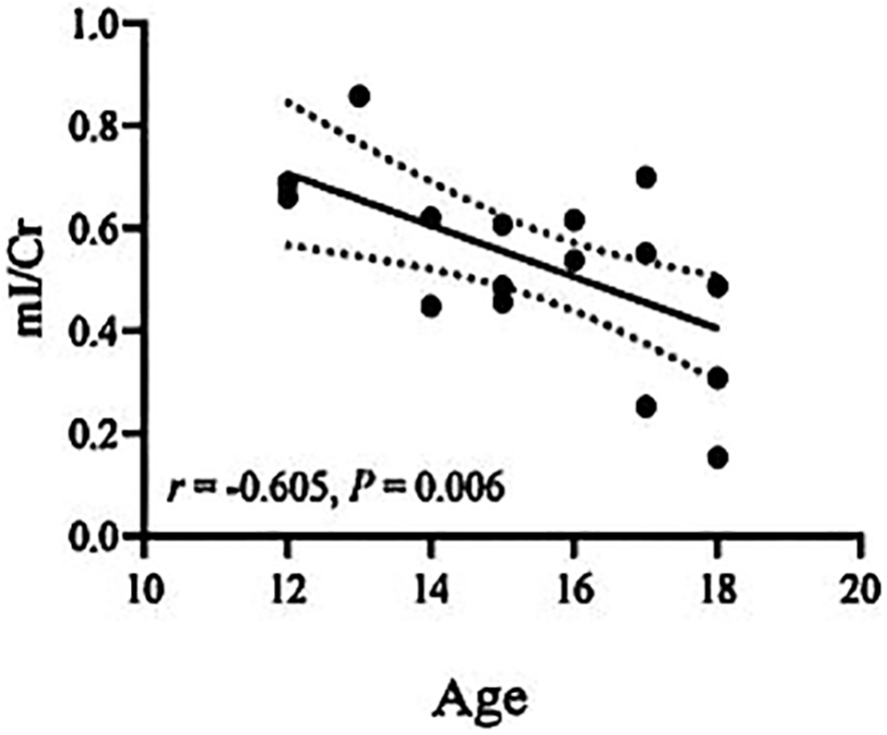
Correlations of mI/Cr ratios in the ventromedial prefrontal cortex (vmPFC) with age in the non-NSSI group in which the band with dotted lines shows 95% confidence interval.

**Figure 3:**
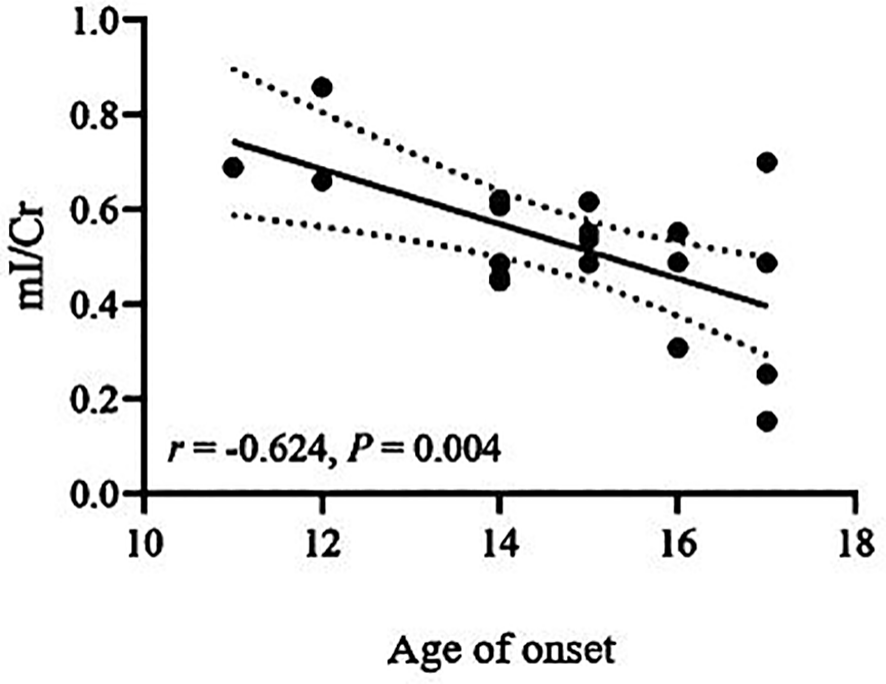
Correlations of mI/Cr ratios in the ventromedial prefrontal cortex (vmPFC) with age of onset in the non-NSSI group in which the band with dotted lines shows 95% confidence interval.

The Cho/Cr ratio and HAMD scores of patients in the NSSI group showed a positive correlation(*r*=0.703,*P<0.00625*), while the mI/Cr ratio of patients in the non-NSSI group negative correlations with age and onset age. The above correlations were statistically significant (*r*=-0.605,*P=0.006;r=0.624,P=0.004*). There was no significant correlation between other indicators (*P>0.05*) (**Table 3**). As detailed in **Figures 1**–**3**.

**Table 3:** Correlations between biochemical metabolite ratios and clinical variables in adolescent patients with bipolar depression with and without NSSI.

	NAA/Cr ratio		Cho/Cr ratio		mI/Cr ratio	
	*r*	*P*	*r*	*P*	*r*	*P*
Age (years)						
NSSI	0.222	0.334	0.346	0.124	-0.145	0.531
Non-NSSI	0.168	0.491	-0.335	0.161	-0.605	0.006
Age of onset (years)						
NSSI	0.090	0.698	0.154	0.504	0.030	0.896
Non-NSSI	0.149	0.542	-0.393	0.096	-0.624	0.004
Duration of illness (months)						
NSSI	0.301	0.185	0.228	0.320	-0.428	0.053
Non-NSSI	-0.011	0.965	-0.016	0.949	-0.214	0.378
HAMD score						
NSSI	0.234	0.308	0.703	<0.001	-0.240	0.294
Non-NSSI	-0.182	0.456	0.310	0.197	-0.066	0.788

NSSI, non-suicidal self-injury; vmPFC, ventromedial prefrontal cortex.

NAA, N-acetylaspartate; Cho, choline; mI=myo-inositol; Cr, creatine.

Bonferroni correction; P < 0.00625 (0.05/8) (significant).

## Discussion

In this study, the age, gender, only child status, residence, education, family history, age of onset, and disease duration of patients with and without NSSI group were no significant differences, which is generally consistent with the results of some previous studies (28–30). The current study found that a history of NSSI in adolescents with BD are each associated with more complex and severe clinical profiles (31). Studies have found that a longer duration of disease (more than 10 years) is a risk factor for NSSI in BD patients (32). Research has found that among adult and adolescent patients with bipolar disorder, female patients are more prone to non-suicidal self-injury compared to male patients (33, 34). However, there is limited research specifically targeting the relationship between adolescent bipolar depression and NSSI. In our previous study, BD patients showed obvious impulsivity. Lin et al. found that impulsivity has a significant impact on NSSI in patients with BD (35). Since NSSI is a fast, effective, and easy-to-implement method by which individuals can regulate negative emotions, individuals with strong impulsivity are more willing to participate in NSSI to obtain the short-term benefits of NSSI (36). Similarly, adolescent patients often lack better coping styles and are more likely to participate in NSSI. Therefore, further attention should be paid to the relationships among coping styles, impulsivity, and NSSI in adolescent patients with bipolar depression. Most of the subjects selected in this study had not received psychotropic drug treatment within one month of entering the study. However, their disease duration was 9 months or even longer. The reasons why these subjects did not receive systematic drug treatment may be related to the following aspects: (1) The subjects themselves and their guardians have insufficient understanding of the disease and its treatment. After the condition is stable or improves, they stop taking the drugs by themselves, and their compliance with treatment is poor; (2) There may be negative effects such as weight gain and metabolic syndrome after taking drug treatment, further reducing the compliance of adolescent patients with medication (37); (3) The economic burden caused by long-term medication and follow-up may make it unaffordable for many families. One patient took the mood stabilizer lithium carbonate within one month before entering this study, and the duration of taking it was less than one week. Currently, the mechanism of action of lithium carbonate in stabilizing mood is unclear. The latest evidence indicates that lithium carbonate enhances glycogen synthase kinase 3 inhibition, changes in homeostatic synaptic plasticity levels, and microRNA expression regulation as the key mechanisms for stabilizing mood, no study has found that lithium carbonate can have a clear impact on neural metabolism (38). At the same time, all the subjects in this study did not take other drugs that could significantly affect the level of neural metabolism.

NSSI stimulates the regions of the brain responsible for regulating depressed mood. Regarding the neurometabolic characteristics of NSSI in adolescent patients with bipolar depression, the present study found that the NAA/Cr ratio in the vmPFC was lower in the NSSI group than in the non-NSSI group. NAA, as a biomarker of neuronal density and survival, is a metabolite synthesized in neural mitochondria and is involved in numerous processes, including mitochondrial energy production, myelin and lipid formation, as well as neuronal protection against osmotic stress. Therefore, the level of NAA is closely related to neuronal function (39). Studies have shown that NAA positively impacts mental health by elevating glutathione levels in the brain (40). Glutathione, being the primary antioxidant in the brain, plays a role in oxidative stress response, offering neuroprotection against stress and stress-related toxicity (41). A decline in NAA levels directly impacts glutathione production, diminishing its neuroprotective capacity and leading to neuronal damage and dysfunction. In adolescent bipolar depression patients, NSSI behavior often signifies an attempt to self-regulate extreme negative emotions, with oxidative stress disorder being linked to NSSI (42). NSSI exerts long-lasting adverse effects on the central nervous system of adolescents with bipolar depression, causing neuronal damage and dysfunction. Olvera et al. conducted a ^1^H-MRS study on the left DLPFC of adolescent patients with BD and HCs and found that NAA/Cr values in the DLPFC of adolescent patients with BD were lower than those in the HC group, which may indicate neuronal dysfunction in this region (24). Jonika et al. also found that BD subjects had lower levels of NAA/Cr values (43). Previous studies have consistently reported lower NAA/Cr values in the prefrontal cortex of BD patients. Currently, no studies have revealed that mood stabilizers and antipsychotics significantly impact the levels of neurometabolites in the brains of patients with bipolar disorder (44, 45). However, it’s noteworthy that N-acetylcysteine (NAC), serving as both a prescription drug and a nutritional supplement, has the ability to regulate the excitatory neurometabolite glutamate in the brain. Recently, NAC has been explored as a potential treatment for patients suffering from various mental illnesses (46). With its potential to mitigate stress-induced oxidative damage and glucose-mediated neurotoxicity, NAC may prove beneficial in the treatment of NSSI (47). This study compared the NAA/Cr values in the vmPFC brain region between adolescent bipolar depression patients with and without non-suicidal self-injury (NSSI), indirectly exploring the potential relationship between NSSI and neuronal functional status. In future research, neuronal dysfunction may emerge as a reliable neurometabolic characteristic of NSSI in adolescent bipolar depression patients, warranting further investigation.

We also found no significant differences in the mI/Cr or Cho/Cr values between the NSSI group and the non-NSSI group. mI is found in glia and participates in the phosphoinositose cycle. When the synthesis of phosphoinositol by mI is blocked, the mI level increases, and the release of excitatory neurotransmitters (triggered by phosphoinositol) decreases, leading to the occurrence of depression. Tundo et al. found that there was no significant difference in mI levels in the anterior cingulate cortex of BD patients (48). Patel et al. found that compared with the HC group, patients with bipolar depression had increased mI/Cr values in the ventrolateral PFC (49). The variations in results could be attributed to factors like the limited sample size in these studies and alterations in the parameters of the evaluation tools. Hence, it is imperative to augment the sample size for subsequent investigations. Studies have demonstrated increased Cho/Cr values in the basal ganglia of BD patients, but there is no consistent evidence that Cho/Cr values change in other regions of the brain in BD patients (50, 51). Cho serves as a marker for cell membrane integrity. It is intimately linked to the breakdown and synthesis of membrane phospholipids, reflecting the renewal of the cell membrane. Under pathological conditions, the rupture of nerve cell membranes can result in an elevation of Cho content. Currently, research findings regarding Cho levels in patients with BD and NSSI are inconsistent. Based on this, our research results can provide a reference for researchers to conduct studies on Cho in the brain. There are also some inconsistencies between previous findings and our current findings. Zhang et al. found that adolescent NSSI is associated with thalamic dysfunction and abnormal NAA and Cho metabolism (25). However, we did not find any significant differences in Cho/Cr values between the two patient groups in our study. Through the aforementioned research, we discovered that the current research findings lack consistency. This inconsistency may be attributed to various factors: (1) the age, disease duration, clinical condition, and medication status of the subjects; (2) variations in magnetic resonance field strength, technical parameters, and image data processing; (3) the relatively small sample size, which makes it challenging to detect minor changes in metabolite concentration. The prevention and diagnosis of NSSI in adolescents with bipolar depression is difficult when based on subjective recall and descriptions of patients and their families, as there is a lack of clear diagnostic features or biomarkers. Identifying neurobiological markers associated with symptoms, along with delivering higher-quality clinical diagnoses, holds significant importance for the early screening and diagnosis of adolescent bipolar depression patients who engage in non-suicidal self-injury.

In this study, we found a positive correlation between the Cho/Cr values and HAMD scores in the NSSI group, while there were negative correlations of the mI/Cr values with age and onset age in the non-NSSI group. This suggests that the level of membrane phospholipid breakdown metabolism in vmPFC of NSSI patients increases with the severity of depression, indicating that NSSI in adolescent bipolar depression patients may be associated with membrane phospholipid breakdown metabolism in vmPFC. Acetylcholine is decomposed by acetylcholinesterase to produce Cho, which can be re-uptaken back into neurons and used again as a raw material for synthesizing acetylcholine. This forms a cycle, ensuring the effective utilization of neurotransmitters and the normal transmission of neural signals. Some studies have found that age is negatively related to the activity of acetylcholine transferase in cholinergic neurons, indicating that the change in Cho levels may be related to age (52). However, we did not find a correlation between Cho levels and age in our study. Our research findings differ from those mentioned above, possibly due to variations in our research samples. While both studies encompassed patients with bipolar disorder, our study specifically focused on adolescent bipolar depression patients who also exhibited non-suicidal self-injury. Zhong et al. found that the Cho/Cr values in the prefrontal lobe of BD patients was not significantly correlated with age and onset age (53), which is largely consistent with our study. We found that the mI/Cr values decreased with age and onset age in the non-NSSI group, but we found no correlations of the mI/Cr ratio with age and onset age in the NSSI group. This suggests that the alterations in mI within the vmPFC brain region among patients exhibiting non-suicidal self-injury behaviors may remain unaffected by age and age of onset. Further research is warranted to increase the sample size. It is of great significance to clarify the neurometabolic mechanisms underlying NSSI in adolescent bipolar depression patients, and this warrants further investigation. Future studies should delve deeper into the pathogenesis and influencing factors of NSSI in this patient population, aiming to gain a more comprehensive understanding of the neurometabolic characteristics and potential risk and protective factors associated with NSSI behaviors. This, in turn, can provide objective evidence for early detection and diagnosis.

### Limitations

To our knowledge, the present study is the first to use voxel ^1^H-MRS technology to compare vmPFC neural metabolites between adolescent patients with bipolar depression with NSSI and those without NSSI. The advantage of our study is that we selected adolescent patients with bipolar depression, thereby avoiding confounding effects of ageing on biochemical metabolic values. In addition, the present study used the single voxel ^1^H-MRS technique, which can detect more biochemical metabolic values than the multivoxel ^1^H-MRS technique and has a more stable spectral baseline. However, there are some limitations in this study, as follows:(1) This is a cross-sectional study. Participants were examined solely based on their current emotional state and behavior, without longitudinal tracking to observe how neural metabolism fluctuates with changes in emotions and behaviors. In the future, further follow-up studies can be conducted to investigate the relationship between NSSI relief and neurometabolic changes in adolescent bipolar depression patients. (2) Due to the small sample size and the absence of a healthy control group, it is impossible to analyze the differences in vmPFC metabolites between adolescent bipolar depression patients with NSSI and healthy control groups. In the future, we can expand the sample size and establish a healthy control group for further analysis.

## Conclusions

Despite the limitations mentioned above, our study still provides valuable clinical data. We found a decrease in NAA metabolism in the vmPFC of adolescent patients with bipolar depression and NSSI, indicating that NSSI in adolescent patients with bipolar depression may be related to vmPFC neuronal dysfunction. In conclusion, the changes in NAA can provide an objective and observable neurometabolic indicator for the early diagnosis of non-suicidal self-injury behaviors in adolescent bipolar depression patients. In addition, the severity of depression in adolescent patients with bipolar depression and NSSI may be related to the membrane phospholipid catabolism in the vmPFC. In the future, we will expand the sample size and conduct prospective cohort studies to further elucidate potential neurometabolic biomarkers linked to NSSI in adolescents with bipolar depression, thereby providing a basis for the early identification and diagnosis of patients exhibiting NSSI.

## Data Availability

The original contributions presented in the study are included in the article/supplementary material. Further inquiries can be directed to the corresponding author.
